# Supplementary material for: Splice-Junction-Based Mapping of Alternative Isoforms in the Human Proteome
Source: Cell Rep. Author manuscript; Available in PMC 2020 Jan 15. (PMC6961840; doi:10.1016/j.celrep.2019.11.026)
Supplement: 2 [file NIHMS1546469-supplement-2.zip › DF1/PXD010154/Liver/Liver_7_CAMSAP3_ASPAAPADGAAPAQPSHAIAFCLK.pdf]

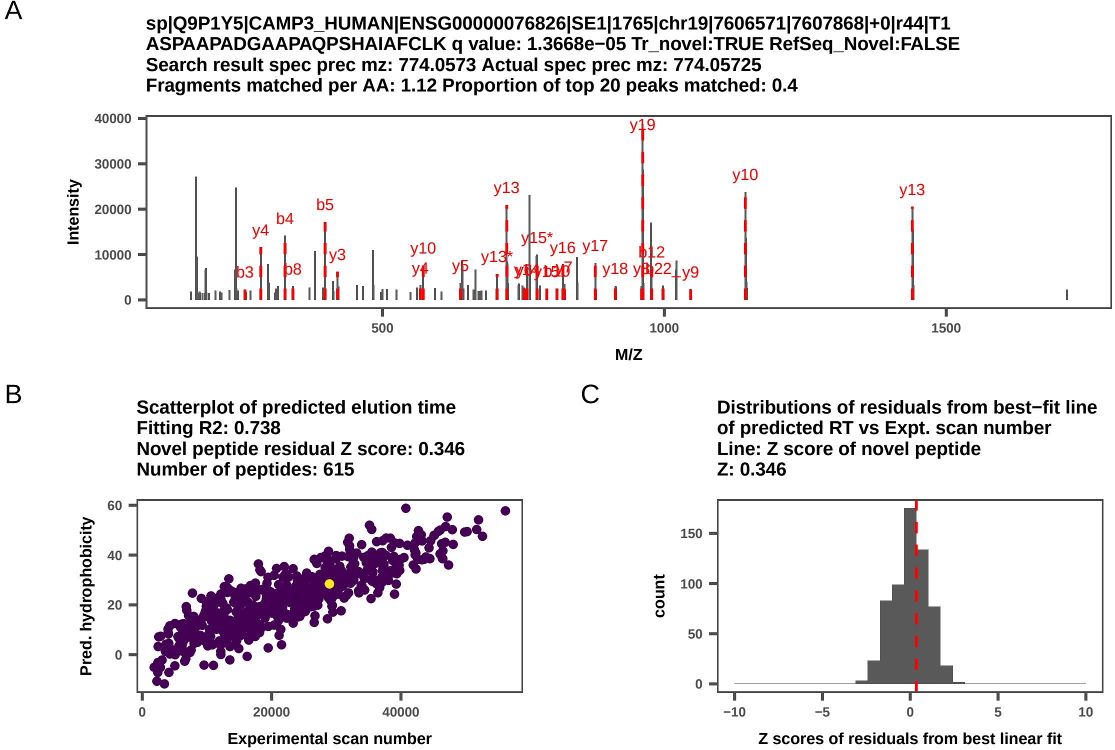
A
sp|Q9P1Y5|CAMP3_HUMAN|ENSG00000076826|SE1|1765|chr19|7606571|7607868|+0|r44|T1
ASPAAPADGAAPAQPSHAIAFCLK q value: 1.3668e−05 Tr_novel:TRUE RefSeq_Novel:FALSE
Search result spec prec mz: 774.0573 Actual spec prec mz: 774.05725
Fragments matched per AA: 1.12 Proportion of top 20 peaks matched: 0.4
Intensity
40000
30000
20000
10000
0
M/Z
500
1000
1500
y19
y10
y13
y13
b5
b4
y4
y3
b3
b8
y10
y4
y5
y13*
y15*
y16
y17
y18
b12
y9
B
Scatterplot of predicted elution time
Fitting R2: 0.738
Novel peptide residual Z score: 0.346
Number of peptides: 615
Pred. hydrophobicity
60
40
20
0
0
20000
40000
Experimental scan number
C
Distributions of residuals from best−fit line
of predicted RT vs Expt. scan number
Line: Z score of novel peptide
Z: 0.346
count
150
100
50
0
−10
−5
0
5
10
Z scores of residuals from best linear fit
